# Primary Extraosseous Ewing sarcoma of the Rectosigmoid region in an adult male: a rare case report

**DOI:** 10.1093/omcr/omaf263

**Published:** 2025-12-26

**Authors:** Utsav Nath Adhikari, Oshan Shrestha, Abhinav Kumar Singh, Supriya Upreti, Sameen Khatiwada, Dipak Kumar Yadav

**Affiliations:** Department of Pathology, Nobel Medical College Teaching Hospital, Kanchanbari Road, Biratnagar-56613, Morang District, Koshi Province, Nepal; Department of Pathology, Nobel Medical College Teaching Hospital, Kanchanbari Road, Biratnagar-56613, Morang District, Koshi Province, Nepal; Department of Pathology, Nobel Medical College Teaching Hospital, Kanchanbari Road, Biratnagar-56613, Morang District, Koshi Province, Nepal; Department of Pathology, Nobel Medical College Teaching Hospital, Kanchanbari Road, Biratnagar-56613, Morang District, Koshi Province, Nepal; Department of Radiology, Nobel Medical College Teaching Hospital, Kanchanbari Road, Biratnagar-56613, Morang District, Koshi Province, Nepal; Department of Surgery, Nobel Medical College Teaching Hospital, Kanchanbari Road, Biratnagar-56613, Morang District, Koshi Province, Nepal

**Keywords:** extraosseous ewing sarcoma, rare, rectosigmoid region, small round blue cell tumor

## Abstract

Background: Ewing sarcoma rarely arises in the gastrointestinal (GI) tract, and rectosigmoid presentations are exceptionally uncommon. Case presentation: 36-year-old man with rectosigmoid mass showing small round blue cells with pseudorosettes, CD99 and FLI1 positive, diagnosed as Ewing sarcoma; managed with surgical resection followed by chemotherapy. Conclusion: Primary EES of rectosigmoid is extremely rare; diagnosis requires morphology, IHC and molecular testing wherever possible; early biopsy allows neoadjuvant chemo before surgery.

## Introduction

Extraosseous Ewing sarcoma comprises about 30% of Ewing sarcoma family tumors and typically arises in soft tissue, rarely in the GI tract [1]. Only 7 cases of colorectal Ewing sarcoma has been reported out of which 5 are in the left colon [2]. Patients are often in their 30s–40s and present with abdominal pain or a palpable mass [3, 4]. EES is frequently misdiagnosed as GIST or lymphoma on imaging due to overlapping features [5]. Accurate diagnosis hinges on histology, immunostaining (e.g. CD99, FLI1), and molecular confirmation of EWSR1 rearrangement [5, 6]. We present a rare rectosigmoid EES case emphasizing diagnostic and treatment considerations.

## Case presentation

A 36-year-old man presented with a 3-month history of intermittent lower abdominal discomfort and a gradually enlarging, firm, mobile mass in the right lower quadrant without peritoneal irritation or obstruction.

**Figure 1:**
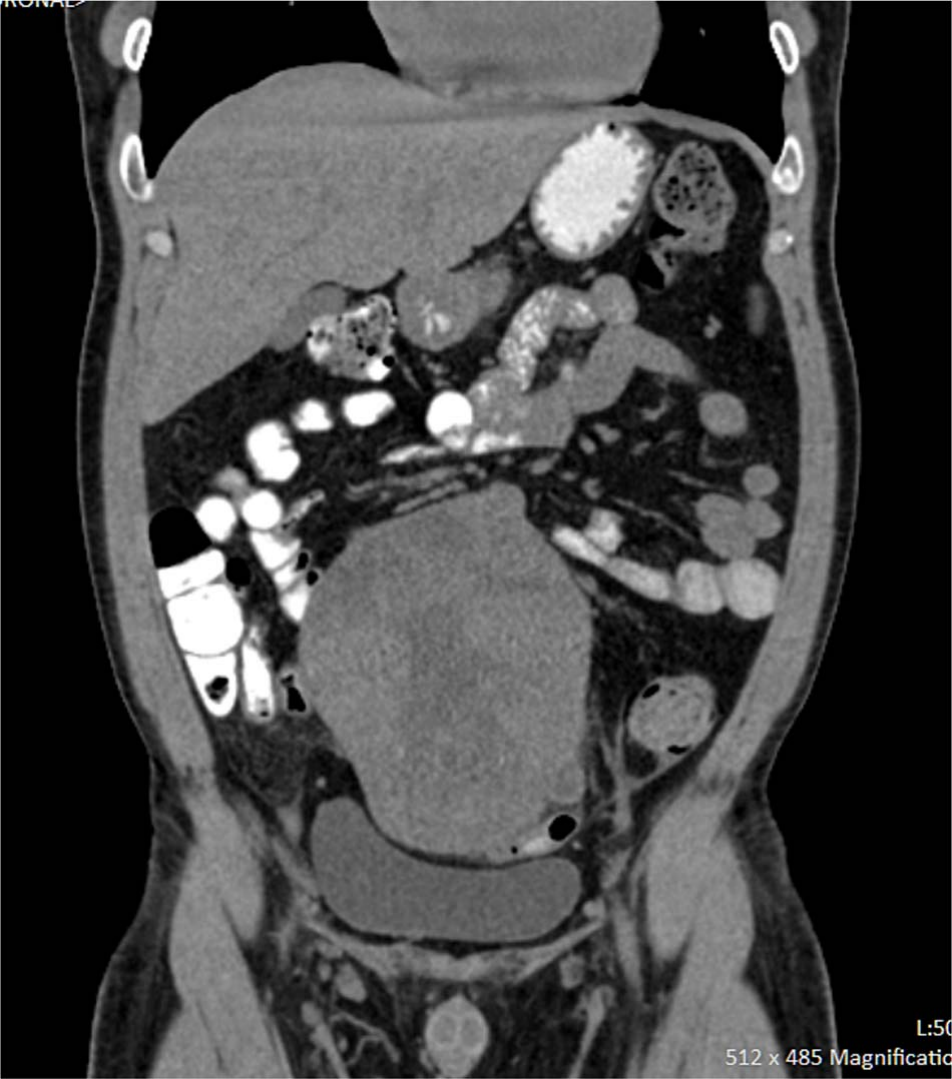
CT scan showing a lobulated enhancing mass arising from the rectosigmoid region with focal necrosis.

On imaging, ultrasound showed a well-circumscribed lower abdominal mass, and contrast-enhanced CT revealed a lobulated, heterogeneously enhancing mass from the medial rectosigmoid wall, initially suspected as GIST (Figure 1).

The patient underwent near-total tumor resection with segmental sigmoid and upper rectum resection and colostomy. Intraoperatively, a 15 × 12 cm mass from the mesenteric sigmoid extending to the lower rectum was noted. Surgery lasted 2 h with approximately 400 ml blood loss and no complications.

On gross, the colonic mucosa was unremarkable; however there was a tumor arising from the wall of sigmoid colon. The tumor measured 14 × 10 × 8 cm, tan-white and fleshy, soft, friable. Cut surface was heterogenous with extensive necrosis and hemorrhage.

**Figure 2:**
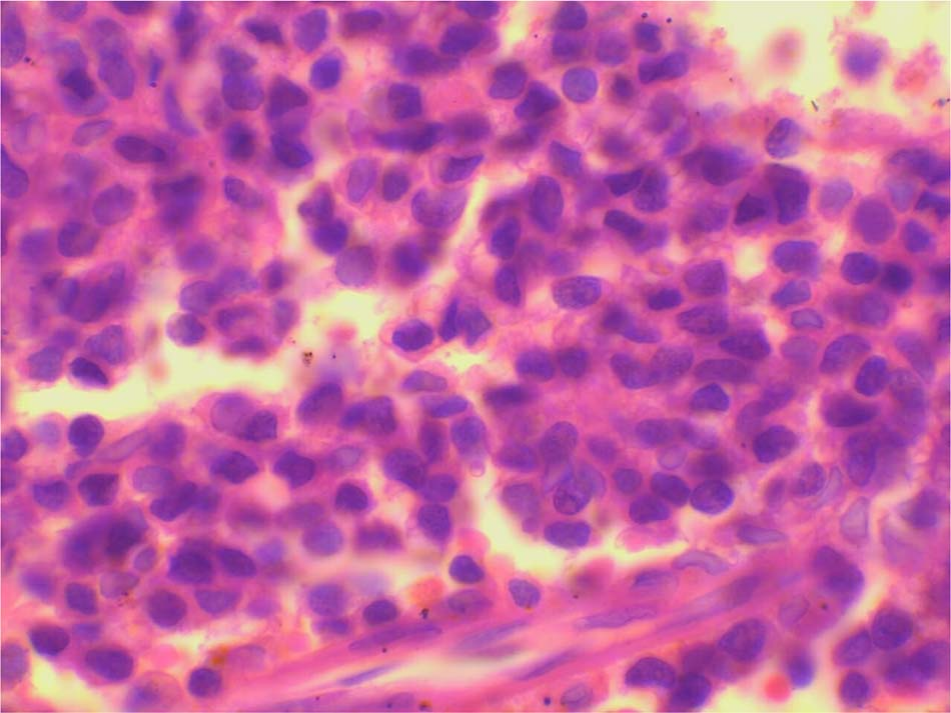
H&E (×400): Small round tumor cells with scant cytoplasm and hyperchromatic nuclei forming pseudorosettes.

On microscopic examination sheets of small round blue cells with hyperchromatic nuclei, scant cytoplasm, pseudorosette arrangements along with atypical mitoses and areas of necrosis were seen (Figure 2). The overlying epithelium was normal.

**Figure 3:**
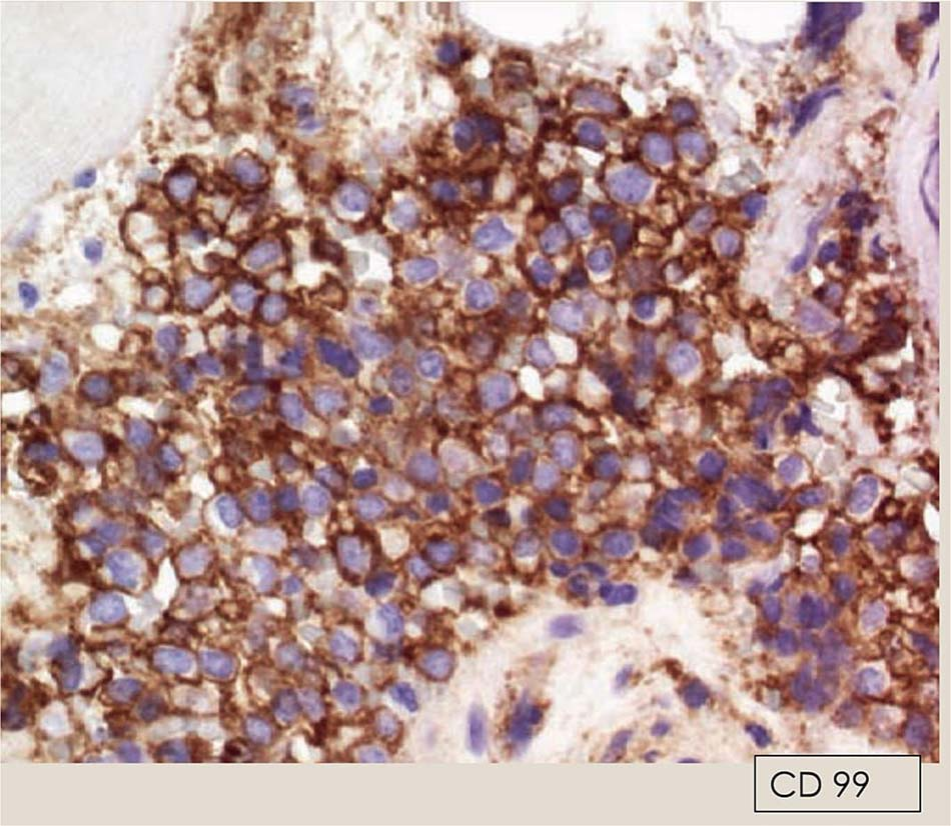
IHC: Strong membranous CD99 staining in tumor cells.

**Figure 4:**
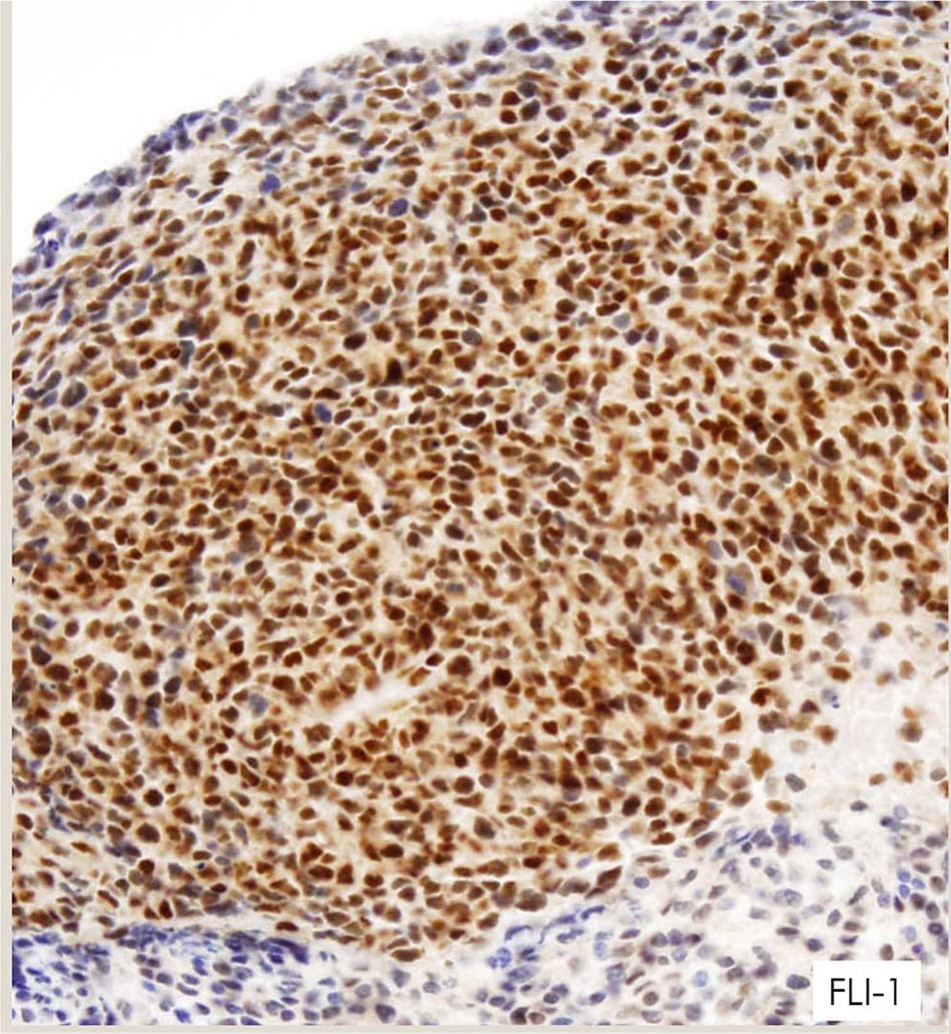
IHC: Strong nuclear FLI1 staining in tumor cells.

Immunohistochemistry showed strong membranous CD99 positivity and nuclear FLI-1 positivity (Figure 3 and 4); negative staining for CD45, cytokeratin AE1/AE3, desmin, and myogenin.

Postoperative recovery was uneventful. The patient commenced Vincristine, Adriamycin, Cyclophosphamide, Ifosfamide, Etoposide chemotherapy (VAC/IE). At six-month follow-up, imaging revealed no recurrence. Molecular confirmation of EWSR1 rearrangement was not performed and is a limitation of this report.

## Discussion

Gastrointestinal EES is rare, especially in the rectosigmoid region [3, 4]. A multicenter review reported small bowel involvement as most common, with only a handful of colonic or rectal origins [3]. Patients often present between ages 30–50 with nonspecific symptoms as in our case [3, 5]. Radiologically, EES can mimic GIST or lymphoma [5].

Histologically, EES is characterized by small round blue cells with scant cytoplasm and necrosis [4]. This can also mimic lymphoma (CD45+), poorly differentiated carcinoma (cytokeratin+), GIST (DOG1/CD117+), and rhabdomyosarcoma (desmin/myogenin+). IHC analysis is thus warranted for confirmation of the diagnosis. For Ewing sarcoma, CD99 expression is sensitive but not specific and must be interpreted alongside FLI1, which improves specificity. In our case we ruled out other close histological mimickers through IHC analysis. Molecular confirmation, whenever feasible, typically detecting EWSR1–FLI1 fusion, is further confirmatory for diagnosis [6].

Management is extrapolated from skeletal Ewing sarcoma protocols: multimodal therapy with surgery, chemotherapy, and occasionally radiotherapy [1]. But there was no preoperative tissue diagnosis in our case and it was operated on line of GIST. The patient thus did not receive any neoadjuvant chemotherapy which could potentially reduce the tumor bulk and potentially reduce postoperative morbidity in our patient since Ewing sarcoma is known to be a highly chemosensitive tumor [7]. Prognosis is best in localized disease (5-year overall survival ~ 60%–70%) and depends on stage, size, and resection status. Because recurrence risk is highest in the first 2–3 years, close clinical and imaging follow-up is recommended for at least 5–10 years [8].

## Conclusion

Primary EES of the rectosigmoid colon is exceedingly rare (only 7 case reports in colorectal region till date), yet should be considered in the differential diagnosis of small round blue cell tumors. Preoperative tissue diagnosis can encourage the bulk of tumor reduction with the help of neoadjuvant chemotherapy which can be further operated for complete resection. Precise diagnosis mandates coordinated morphological evaluation, targeted immunohistochemistry (CD99, FLI1), and molecular confirmation.
